# Hsp90: A New Player in DNA Repair?

**DOI:** 10.3390/biom5042589

**Published:** 2015-10-16

**Authors:** Rosa Pennisi, Paolo Ascenzi, Alessandra di Masi

**Affiliations:** 1 Department of Sciences, Roma Tre University, Viale Guglielmo Marconi 446, Roma I-00146, Italy; E-Mails: rosa.pennisi@uniroma3.it (R.P.); ascenzi@uniroma3.it (P.A.); 2 Istituto Nazionale di Biostrutture e Biosistemi, Viale Medaglie d’Oro 305, Roma I-00136, Italy

**Keywords:** base excision repair, DNA damage response, DNA double strand break, DNA repair, Hsp90, Hsp90 inhibitors, mismatch repair, translation synthesis

## Abstract

Heat shock protein 90 (Hsp90) is an evolutionary conserved molecular chaperone that, together with Hsp70 and co-chaperones makes up the Hsp90 chaperone machinery, stabilizing and activating more than 200 proteins, involved in protein homeostasis (*i.e.*, proteostasis), transcriptional regulation, chromatin remodeling, and DNA repair. Cells respond to DNA damage by activating complex DNA damage response (DDR) pathways that include: (i) cell cycle arrest; (ii) transcriptional and post-translational activation of a subset of genes, including those associated with DNA repair; and (iii) triggering of programmed cell death. The efficacy of the DDR pathways is influenced by the nuclear levels of DNA repair proteins, which are regulated by balancing between protein synthesis and degradation as well as by nuclear import and export. The inability to respond properly to either DNA damage or to DNA repair leads to genetic instability, which in turn may enhance the rate of cancer development. Multiple components of the DNA double strand breaks repair machinery, including BRCA1, BRCA2, CHK1, DNA-PKcs, FANCA, and the MRE11/RAD50/NBN complex, have been described to be client proteins of Hsp90, which acts as a regulator of the diverse DDR pathways. Inhibition of Hsp90 actions leads to the altered localization and stabilization of DDR proteins after DNA damage and may represent a cell-specific and tumor-selective radiosensibilizer. Here, the role of Hsp90-dependent molecular mechanisms involved in cancer onset and in the maintenance of the genome integrity is discussed and highlighted.

## 1. Introduction

Cellular protein homeostasis, also termed proteostasis, regulates protein folding and functions in order to maintain the well-being condition of both the cells and the organism; indeed, protein misfolding and unfolding are associated to several human disease [1]. Molecular chaperones and protein remodeling factors maintain proteostasis, mitigating the life-threatening effects of endogenous and exogenous stressors on the proteome [2].

Heat shock protein 90 (Hsp90) is an evolutionary conserved molecular chaperone that, together with Hsp70 and co-chaperones, makes up the Hsp90 chaperone machinery, which stabilizes and activates more than 200 proteins in mammalian cells [3,4,5,6,7]. Two major cytoplasmic isoforms of Hsp90 are known, the inducible/major form Hsp90α and the constitutive/minor form Hsp90β [8]. One of the main structural differences is that the α form readily dimerizes, whereas the β form undergoes dimerization with much less efficiency (see paragraph 3). Since the isolation of Hsp90 isoforms is rather difficult, most studies have been carried out with the mixture of α and β Hsp90 isoforms [9].

Hsp90 is one of the most abundant and conserved molecular chaperones playing an essential role in eukaryotic cells proteostasis. Indeed, Hsp90 is involved in cellular homeostasis, transcriptional regulation, chromatin remodeling, and DNA repair. The ability of the Hsp90 chaperone machine to correlate protein assembly to protein degradation represents a quality control mechanism and provides plasticity for dynamic protein complexes [10,11].

Differently from other chaperones, Hsp90 is not required for *de novo* protein folding, but rather facilitates the final maturation of specific proteins termed clients. Indeed, most Hsp90 clients must be properly folded in order to interact successfully with their binding partners. Therefore, the Hsp90 chaperone machinery plays a key role in orchestrating the spatial and temporal order of protein interactions [2,3,12]. To ensure the proper protein assembly, the Hsp90 chaperone machinery performs three main functions under normal conditions: (i) it specifically interacts with a vast array of clients through adapter co-chaperones (*e.g.*, p23 and Cdc37); (ii) it stabilizes specific folding intermediates that allows clients to interact with specific binding partners; and (iii) it regulates the ubiquitin-mediated proteasome degradation [2,10,11,13,14].

The Hsp90 activity is regulated at several levels, including the ATPase cycle, the association with conformation-specific co-chaperones, and post-translational modifications [15,16]. Co-chaperones modulate client protein recognition by Hsp90, regulate the ATPase activity of Hsp90, and modulate the client biochemical activities. Among the 20 co-chaperons identified, so far the most important are Cdc37, playing a pivotal role in cell cycle regulation, p23, being a major player in the DNA repair processes, and Aha1, activating the Hsp90 ATPase activity [17,18]. Hsp90 is one of the major eukaryotic phosphoprotein, changes in its phosphorylation status having a deep impact on the chaperone functions [19,20].

Under physiological conditions, Hsp90 represents ~1%–2% of the total cellular protein content, being pivotal to buffer proteostasis against environmental stress [21,22]. Under extreme environmental conditions, the chaperone reservoir can be rapidly exhausted with consequent changes in the functions of Hsp90 clients, thus influencing human health, diseases onset, and evolutionary processes [2]. Because Hsp90 is the molecular chaperone of numerous oncoproteins, it is considered a crucial facilitator of oncogene addiction and represents a validated anti-cancer drug target (see paragraph 2) [23,24]. Indeed, inhibition of the ATPase activity of Hsp90 impairs client protein recognition, thus causing clients degradation [10,25,26].

## 2. Hsp90 and Cancer

Hsp90 is implicated in many pathological conditions, such as ischemia, reperfusion, infections, neurodegenerative diseases, and particularly cancer [27,28,29,30,31,32,33,34]. Indeed, it is well known that many Hsp90 clients are: (i) oncogenic proteins (*e.g.*, EGFR and Her2/HerbB2); (ii) signaling proteins (*e.g.*, Akt/PKB, Raf1, IKK, p53, v-Src, and HIF-1α); (iii) cell cycle regulators (*e.g.*, Cdk4/6); (iv) chimeric signaling proteins (*e.g.*, Bcr-Abl); and (v) hormone receptors (*e.g.*, estrogen and glucocorticoid receptors) [35,36,37,38,39,40,41].

The essential functions of chaperones are subverted during oncogenesis, contributing to malignant transformation and to the rapid somatic evolution. The increased expression of one or more chaperones above the level observed in normal tissues is a common feature of both solid tumors and hematological malignancies [30,33,42]. The increased abundance of chaperones in advanced cancers reflects an appropriate cytoprotective stress response to the hostile hypoxic, acidotic, and nutrient-deprived microenvironment. At the molecular level, the increased chaperone activities allows tumor cells to cope with the imbalanced signaling that is associated with neoplastic transformation, and thereby escape the apoptotic death that would normally ensue. Indeed, the impairment of apoptotic signaling is a common characteristic of cancer cells, since it facilitates survival and expansion thus rendering cells independent of normal regulatory factors and resistant to host defense mechanisms and both chemotherapy and radiotherapy. Moreover, the ability of cancer cells to repair DNA allows them to survive from the DNA damage induced by chemotherapeutic agents or radiation [30,33,42].

In cancer cells, Hsp90 and its co-chaperones form a super-chaperone complex, *i.e.*, the “activated state” of the protein, and the client proteins display a stable association with Hsp90. By contrast, under normal conditions clients interact with low-affinity with Hsp90 since the super-chaperone complex is not formed, Hsp90 being in the so-called “latent state” [43]. The expression of Hsp90 is 2- to 10-fold higher in tumor cells than normal cells [44]. Therefore Hsp90 has become an attractive therapeutic target in cancer treatment, since its inhibition can induce the degradation of clients and promoting enhanced host natural killer cell-mediated tumor killing [41,45,46,47].

Hsp90 levels significantly increase in medulloblastoma [48] and in breast cancer (particularly in ductal carcinomas) [49]. In colorectal cancer, Hsp90 promotes epithelial to mesenchymal transition, invasion, and migration, both *in vitro* and *in vivo*, because of HIF-1α or NF-κB overexpression [50]. Moreover, Hsp90 is an essential regulator of EphA2 stability and signaling [51], which is highly expressed in several cancer cells and is recognized by the host as a self-protein, thus limiting the ability of CD8+ T cells to recognize and kill the tumor. Noteworthy, the 17-DMAG-dependent inhibition of Hsp90 affects EphA2 stability, improving the *in vivo* anti-tumor activity of Hsp90 [52].

Pre-clinical studies attest the potential of Hsp90 inhibition in inducing tumor growth inhibition, in reducing metastatic potential, and in sensibilizing tumors to the effect of other therapies, possibly as a consequence of the inhibition of DSBs repair and cell cycle checkpoint activation [23,33,35,53,54,55,56,57,58,59]. Indeed, the impairment of Hsp90 function has been shown to enhance the cytotoxicity of a variety of chemical and physical DNA damaging agents (*i.e.*, ionizing radiation (IR)), inhibiting, for instance, ATM-dependent repair mechanism [56,60,61]. In particular, the sensitivity of aneuploid cancer cells to Hsp90 inhibition seems to reflect the increased proteotoxic stress associated to the accumulation of misfolded proteins, as supported by the evidence that Hsp90 inhibition potentiates the effects of proteasome inhibitors in multiple myeloma [62,63]. Among several Hsp90 inhibitors under investigation for cancer treatment, almost all target the *N*-terminus or the *C*-terminus of the protein [26]. In particular, inhibitors that bind the N-terminal region containing the ATP pocket represent the most effective anti-cancer drugs (Table 1) [64,65,66,67].

## 3. Structural Aspects of Hsp90 and Related Chaperones

**Figure 1:**
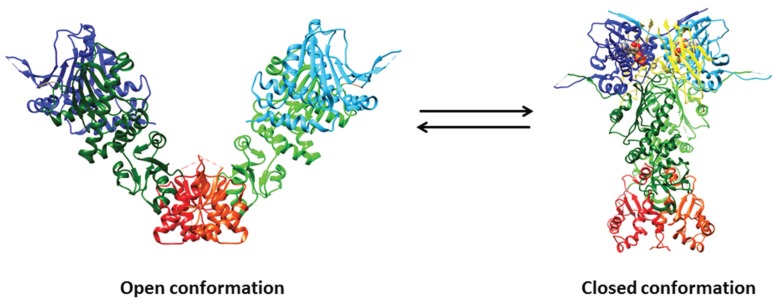
Crystal structures of open (*i.e.*, ligand-free) full-length Hsp90 from *E. coli* (HtpG; PDB ID: 2IOQ) and closed (*i.e.*, ATP- and p23-bound) yeast Hsp90 (PDB ID: 2CG9). The *N*-domain is depicted in blue and cyan, the middle domain in dark green and light green, and the *C*-domain in red and orange. The p23 co-chaperone is in yellow, whereas ATP is depicted through a space-filling representation. Pictures were drawn by UCSF-Chimera [92].

Hsp90 is a homodimeric protein of ~90 kDa displaying ligand-dependent closed and open conformations. Hsp90 and related chaperones are characterized by three structural domains and two disordered regions: (i) the N-terminal domain contains the ATP binding site and is pivotal for dimerization; (ii) the variable, disordered, and charged region named linker, connecting the N-terminal to the middle domain, participates to nucleotide, co-chaperone, and client protein recognition, and finely modulates the Hsp90 activity acting as a “rheostat”; (iii) the middle domain is involved in the hydrolysis of ATP and participates to co-chaperone recognition; (iv) the *C-*terminal domain is responsible for Hsp90 dimerization; and (v) the *C*-terminus unstructured region, ending with the highly conserved Met-Glu-Glu-Val-Asp motif, is involved in the co-chaperone recognition (Figure 1) [29,68].

The structures of the isolated ligand-free and/or ligand-bound N-terminal, middle, and/or *C*-terminal domains of *Escherichia coli* Htpg, yeast (*Saccharomyces cerevisiae*) Hsp90, *Plasmodium falciparum* Hsp90, *Leishmania major* Hsp90, *Trypanosoma brucei* Hsp90, dog Grp94, and human Hsp90 have been determined. The N-terminal domain is a α/β sandwich containing the ATP binding pocket, which extends from the buried face of the anti-parallel β-sheet to the surface (Figure 1). The N-terminal domain of Hsp90 recognizes the natural products geldanamycin and radicicol, inhibiting competitively the hydrolysis of ATP, in a very similar way to ATP. The middle domain of yeast Hsp90 comprises a large α/β/α subdomain at the *N*-terminus, which is bound to a smaller α/β/α subdomain through a series of short α-helices. A hydrophobic patch, containing a highly conserved solvent exposed Trp residue and an amphipathic loop, which has been postulated to recognize client proteins, characterizes the Hsp90 middle domain. Moreover, the Hsp90 middle domain displays a critical loop, containing a highly conserved Arg residue, which is pivotal for the ATPase activity. The *C*-terminal domain of *E. coli* HtpG, displaying a homodimeric structural organization also in the ligand-free protein, is composed by two small mixed α/β domains; the dimerization interface is formed by two α-helices of each subunit which pack together to form a four α-helical bundle (Figure 1) [68].

**Table 1:** Heat shock protein 90 (Hsp90) inhibitor affecting the DNA repair.

Inhibitor	Derivatives	Structure	Pharmacokinetics (nM)	Clinical Study Stage	References
**Geldanamycin** (naturally derived from *Streptomyces hygroscopicus*)		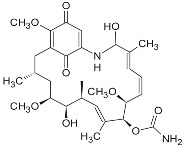	GI_50_ = 1.0 × 10^−1^ nM; LC_50_ = 2.1 × 10^4^ nM; K_d_ = 1.2 × 10^3^ nM	Preclinical	[61,65,69]; www.medchemexpress.net
	**17-AAG (17-allylamino-17-desmethoxygeldanamycin; tanespimycin)**	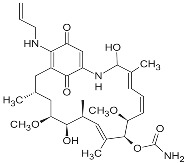	IC_50_ = 5.0 × 10^1^–1.0 × 10^4^ nM	Phase I/II/III	[70,71,72,73,74,75,76,77,78]; www.clinicaltrials.gov; www.medchemexpress.net
	**17-DMAG (17-dimethylaminoethylamino-17-demetoxygelanamycin; alvespimycin)**	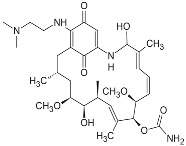	IC_50_ = 6.0 × 10^1^–3.0 × 10^3^ nM; K_d_ = 3.5 × 10^2^ nM	Phase I/II	[72,77,79,80,81]; www.clinicaltrials.gov
**PU-H71**		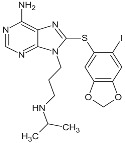	IC_50_ = 5.0 × 10^1^–3.0 × 10^2^ nM	Phase I	[82,83,84]; www.clinicaltrials.gov
**NVP-AUY922**		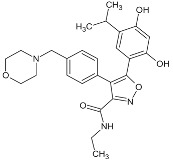	IC_50_ = 1.3 × 10^1^ nM (Hsp90α); IC_50_ = 2.1 × 10^1^ nM (Hsp90β); GI_50_ = 2.0–4.0 × 10^1^ nM; K_d_ = 1.7 nM	Phase I/II	[85,86,87]; www.medchemexpress.net; www.clinicaltrials.gov
**Radicicol** (naturally derived from *Diheterospora chlamydosporia)*		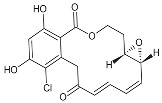	K_d_ = 1.9 × 10^1^ nM; IC_50_ = 2.0 × 10^1^ nM	Preclinical	[65]
**STA9090 (Ganetespib)**		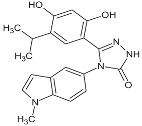	IC_50_ = 1.0–5.0 × 10^1^ nM	Phase I/II/III	[88,89,90,91]; www.clinicaltrials.gov; www.medchemexpress.net

The open (*i.e.*, ligand-free) structure of full-length *E. coli* HtpG and the closed (*i.e.*, ligand-bound) structures of full-length yeast Hsp90, *E. coli* HtpG, and dog Grp94 have been determined. Overall, the structures of the three domains of the full-length proteins are very similar to those of the isolated domains; however, some differences were observed in the *N*-terminal domain (possibly reflecting nucleotide binding and dimerization) and in the α-helical segment that protrudes out from the *C*-terminal domain (Figure 1) [68].

The structure of the open full-length *E. coli* HtpG displays an open V-like conformation. The solvent-exposed hydrophobic patches appear to represent potential binding sites for client proteins, whose recognition by Hsp90 is favored by its flexible conformation. In the ligand-free full-length *E. coli* HtpG, the lid of the ATP binding pocket is positioned in such a way as to block nucleotide binding, representing a case of auto-inhibitory mechanism. A large structural rearrangement(s) of the N-terminal domain relative to the middle domain is pivotal to allow the closure of the lid after nucleotide binding [93].

**Figure 2:**
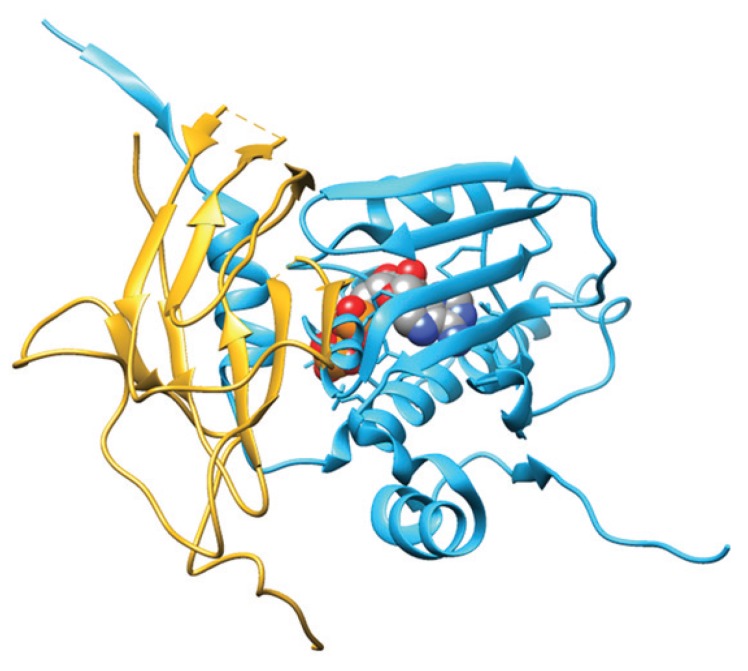
Crystal structure of yeast Hsp90/ATP/p23 closed chaperone complex (PDB ID: 2CG9). The Hsp90 *N*-terminal domain is shown in cyan, the p23 co-chaperone is in yellow, and ATP is depicted through a space-filling representation. Pictures were drawn by UCSF-Chimera [92].

In contrast to the open ligand-free form of full-length *E. coli* HtpG, the closed form of yeast Hsp90 complexed with the AMP-PNP and p23 is relatively compact (Figure 2) and characterized by: (i) the dimerization not only of the *C*-terminal regions, but also of the *N*-terminal domains; (ii) extensive contacts between the domains within each monomer; and (iii) twisting of the subunits. In particular, the ATP binding region undergoes a large movement around a hinge created by two critical Gly residues, thus exposing a hydrophobic patch that contributes to build up the dimerization surface of the N-terminal domains [94]. The two p23 co-chaperones bind into grooves occurring at the interface between the two N-terminal domains of yeast Hsp90, through an ATP-dependent mechanism. Although the inspection of the yeast Hsp90-p23-AMP-PP structure does not directly reveal how p23 inhibits the ATPase activity of Hsp90, it has been postulated that p23 stabilizes the closed dimerized state of full-length Hsp90 preventing the release of ADP and phosphate [94].

## 4. Role of Hsp90 in the Genome Stability Maintenance

Cells respond to DNA damage by activating the complex DNA damage response (DDR) pathway that includes the cell cycle arrest, the transcriptional and post-translational activation of a subset of genes including those associated with DNA repair, and, under some circumstances, the triggering of programmed cell death. The efficacy of the DDR is influenced by the nuclear levels of DNA repair proteins, which are regulated by balancing between protein synthesis and degradation, and the control of nuclear import and export. The inability to repair DNA damage leads to genetic instability, which in turn may enhance the rate of cancer development [95,96,97].

Historically, the Hsp90 molecular chaperone system had been viewed as a strict cytosolic machine [17]. Now it is becoming clear that Hsp90 and its associated co-chaperones have important functions into the nucleus, including chromatin remodeling, DNA transcription, RNA processing, DNA replication, telomere maintenance, and DNA repair [15,98]. Indeed, Hsp90 participates to DNA repair and interacts with DNA metabolic proteins *via* the p23 co-chaperone or upon phosphorylation [15,98,99,100].

### 4.1. The DNA Double-Strand Break Response

Among several types of lesion, the DNA double-strand break (DSB) is one of the most deleterious and harmful. DSBs arise from both endogenous and exogenous sources, including reactive nitrogen and oxygen species, replication errors, chemical mutagens, and IR [101].

**Figure 3:**
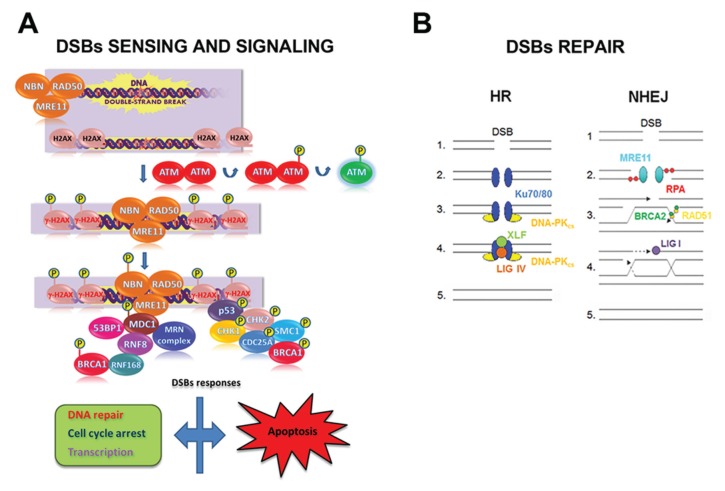
Double-strand break (DSB)s sensing, signaling and repair in mammalian cells. (**A**) Schematic representation of the early steps of DSBs sensing and signaling. After a DSB induction: (i) the MRE11/RAD50/NBN complex, made up of MRE11, RAD50 and NBN, localizes at damage site; (ii) ATM undergoes auto-phosphorylation at the Ser1981 residue, with the consequent dissociation of ATM dimers and ATM activation; (iii) ATM phosphorylates H2AX histone that, as γ-H2AX, serves as a platform for the assembly of proteins involved in DNA repair, cell checkpoint response, and transcription. Depending on the severity of the DNA damage and on the cell type, cells may undergo apoptosis; (**B**) Once the cell has sensed the DSB, the DNA repair machinery is recruited to the lesion in relation to the cell cycle stage. In G1 phase cells undergo repair predominantly through NHEJ repair pathway, whereas in G2/M the presence of replicated DNA allows the repair through the HR pathway. So, during the NHEJ pathway, in the presence of a DSB (1), the broken ends are bound by Ku70/80 heterodimer (2) that recruit DNA-PK_CS_ (3). After the ends have been processed, the XRCC4/Ligase IV complex completes the final ligation step (4) and the damage is repaired (5). On the contrary, in the presence of a DSB (1), the HR pathway requires MRE11 or Exo1 exonuclease activity to resect the DNA ends forming a 3’ overhang; this structure is stabilized by RPA (2) and then loaded on the homologous DSB region by the strand exchange protein RAD51 and by BRCA2 (3), leading to the formation of the Holliday junctions intermediate (4). Endonuclease and resolvase proteins are involved in resolving the Holliday junctons intermediate (5).

DSB elimination can be divided in three phases (*i.e.*, sensing, signaling, and repair) and involves the spatio-temporal orchestration of a high number of proteins. Briefly, the sensing phase includes: (i) DSB recognition by the MRE11/RAD50/NBN complex; (ii) ATM activation; (iii) histone H2AX phosphorylation (the phosphorylated form of H2AX is named γ-H2AX), (*iv*) MDC1 recruitment; and (v) MRE11/RAD50/NBN retention (leading to further ATM activation and γ-H2AX spreading). γ-H2AX, the initial DSB sensor, represents the site for the accumulation of signaling and repair proteins to DNA breaks to form the so-called IR-induced foci (IRIF) [102,103]. After sensing the damage, the signaling phase is activated. Indeed, DSBs trigger a myriad of post-translational modifications that alter catalytic activities and specific protein-protein interactions, followed by the reversal of these changes as the repair is completed (Figure 3).

Two fundamentally different DSB repair pathways, *i.e.,* the non-homologous end-joining (NHEJ) and the homologous recombination (HR), have been identified both in mammalian cells and in yeast [104,105,106,107]. Both NHEJ and HR DSB repair pathways may be activated simultaneously and cooperatively to repair DNA lesions [108,109,110].

NHEJ (i) brings the DNA termini together in a protein-DNA complex; (ii) does not require the presence of an undamaged template; and (iii) is active throughout the whole cell cycle [111,112]. Therefore, NHEJ is considered as a flexible but conservative DNA repair mechanism that enables a direct rejoining of broken DNA termini, although through an error-prone process [108,113]. To initiate NHEJ, the Ku70/80 heterodimer binds to blunt or near-blunt DNA ends. The DSB-Ku70/80 heterodimer complex recruits and activates the DNA-PKcs adduct that triggers an extensive signaling cascade orchestrating downstream repair processes [114]. NHEJ repair is facilitated by the scaffold proteins XRCC4 and XLF (also called Cernunnos) that bind DNA Ligase IV, the enzyme responsible for sealing the break. If DNA ends need nucleolytic processing before ligation, the Artemis endonuclease, a DNA-PKcs-interacting protein, provides this activity (Figure 3) [111,115].

The HR mechanism depends on the use of a template, as can be found on a sister chromatid during the S and G2 cell cycle phase. Since the HR mechanism uses an undamaged DNA template to restore chromosome integrity, it has the potential to repair DSBs more faithfully than NHEJ. The search for sequence homology to repair DNA requires the presence of single-strand DNA at the DSB end [97,111,116]. This intermediate can be generated by the nucleolytic degradation of the 5’ strand of a DSB end in a process mediated by the MRE11/RAD50/NBN complex. This complex recruits CtIP and initiates the resection, the EXO1 and DNA2 nucleases perform the bulk of end-resection required for HR. In this process, DNA2 acts in complex with the RecQ helicases BLM and/or WRN (Figure 3) [117].

In mammalian cells, the HR mechanism seems to be less utilized than NHEJ in the repair of DSBs, but defects in the HR mechanism do enhance cellular radio-sensitivity. Since most cells arrest at the G2 phase in response to IR exposure and the HR mechanism mainly works in late-S and G2 phases, inhibiting the HR mechanism would be a good strategy for cancer cells treatment [118].

### 4.2. Hsp90 and the DNA Damage Response Clients

Although the cellular circumstances that incorporate Hsp90 into an optimal DNA damage response to IR remains still unknown, multiple components of the DSBs repair machinery, including BRCA1, BRCA2, RAD51, CHK1, DNA-PKcs, members of the FA pathway, histones, and components of the MRE11/RAD50/NBN complex have been reported to be Hsp90 clients [60,119,120,121,122,123,124,125]. Of note, the Hsp90 inhibitors geldanamycin (a highly cytotoxic natural antibiotic isolated from *Streptomyces hygroscopicus*, which binds Hsp90 in the ATP-binding site) [4,65,69,126], its derivates 17-(allylamino)-17-demethoxygeldanamycin (17-AAG) [26,70,127] and 17-dimethylaminoethylamino-17-demethoxygeldanamycin (17-DMAG) [59,71,72,79,127,128], PU-H71 (a member of a class of inhibitors with purine scaffold) [129,130], NVP-AUY922 (a member of a class of inhibitors possessing a pyrazole scaffold) [85,127,131,132], ganetespib (also named STA-9090) [88], and radicicol [65,133] affect the DNA repair, thus highlighting the pivotal role of Hsp90 in the DDR (Table 1) [25,26,57,60,134,135,136].

#### 4.2.1. BRCA1, BRCA2, and RAD51

BRCA1 is a nuclear tumor suppressor critical for DSBs and inter-strand cross-links by the HR mechanism [137]. BRCA1 is phosphorylated by ATM, ATR, and CHK2 kinases in response to DNA damage, then phosphorylated BRCA1 organizes multiple distinct protein complexes that recognize and repair damaged DNA and activate cell cycle checkpoints [138,139]. Except for DNA-PKcs, the activity and/or recruitment of BRCA2, CHK1, FANCA, the MRE11/RAD50/NBN complex, and RAD51 to the DSBs is BRCA-dependent [139,140,141,142,143].

Among the proteins involved in the HR mechanism and sensitive to Hsp90 inhibitors, BRCA1 appears to be the most upstream, being sensitive to the Hsp90 inhibitor 17-AAG [124]. Since tumor cells expressing high levels of BRCA1 are resistant to both IR and several classes of chemotherapeutic agents, the ablation of BRCA1 expression through Hsp90 inhibitors may restore sensitivity to anti-tumor agents, thus representing a possible cancer therapeutic strategy [124,144]. Moreover, the intrinsic sensitivity of BRCA1-mutant or -deficient cells to 17-AAG suggests that this agent might also show efficacy in both primary BRCA1 mutant tumors as well as in sporadic tumors that have lost BRCA1 expression by non-mutational means [124].

BRCA2 directly interacts with Hsp90, 17-AAG causing BRCA2 degradation [57]. Since BRCA2 promotes RAD51-mediated HR at sites of DSBs [145], 17-AAG-dependent BRCA2 degradation leads to RAD51 impairment and induces a delay in the IR-induced BRCA2-mediated RAD51 foci formation. After irradiation, RAD51 foci formation is also inhibited by the treatment of cancer cells with PU-H71 and NVP-AUY922 [136,146]. Both non-irradiated and irradiated cells treated with ganetespib are characterized by the degradation of RAD51 and by a lowered activation of the ATM kinase and other proteins involved in DNA repair [89]. The delay in RAD51 focal assembly leads to a decrease in the rate of the successful HR mechanism. In turn, the persistence of unrepaired DNA damage may increase the time over which cells trigger apoptosis because of DSBs. Moreover, the persistence of unrepaired DNA damage combined with CHK1 depletion (see paragraph 4.2.2) is also likely to increase the probability of an attempted cell cycle progression with an unacceptable/unsafe burden of DNA damage [136].

Since also p53 and CHK1 are Hsp90 clients and interact with RAD51, the 17-AAG-mediated downregulation of these two proteins could be involved in lowering the level of RAD51 [57,147,148].

#### 4.2.2. CHK1

The DNA damage-activated cell cycle checkpoint pathways are evolutionarily conserved signaling pathways that regulate cell cycle progression, programmed cell death, and DNA repair [149]. CHK1 is a key regulator of the signaling pathway activated by DNA replication stress and DNA damage. Indeed, following DNA replication fork stalling, single-stranded regions of DNA (ssDNA) accumulate, RPA binds to ssDNA, and the ATR kinase localizes at the stalled fork together with its binding partner ATRIP [150,151]. The stalled fork also recruits DNA polymerase α, which then participates in the RAD17-dependent recruitment of the PCNA-like RAD9-Hus1-RAD1 clamp complex to chromatin. Once bound to chromatin, the RAD9-Hus1-RAD1 complex facilitates the ATR-mediated phosphorylation and activation of CHK1 [152].

CHK1 performs several functions that support cell survival. Indeed, CHK1 (i) promotes cells arrest in G2; (ii) slows the progression through the S phase by inhibiting the initiation of new replication sites and stimulating the firing of dormant origins placed in proximity of stalled forks; and (iii) avoids fork collapse into DSBs. At the molecular level, CHK1 is activated at stalled replication forks, the nucleoplasm representing the site where CHK1 phosphorylates target proteins such as Cdc25C and Cdc25A, two cell cycle phosphatases that in turn activate the Cdk1-cyclin B complex. Because Cdc25A is also required for the activation of Cdk2 complexes, which then control the firing of origins of replication, the activation of this pathway blocks the S phase progression [152]. Correspondingly, the disruption of the CHK1 signaling pathway is associated with increased sensitivity to genotoxins [153]. Additionally, cells in which CHK1 has been deleted by gene targeting are sensitive to the replication inhibitor aphidicolin and IR [154].

CHK1 is an Hsp90 client, indeed the 17-AAG Hsp90 inhibitor leads to CHK1 degradation and to the disruption of the CHK1-mediated Cdc25A degradation. Overall, Hsp90-dependent modulation of CHK1 stability highlights a critical role played by this kinase in maintaining cell viability following replication stress. Of note, the disruption of CHK1 function(s) by 17-AAG sensitize tumor cells arrested in the S phase by gemcitabine treatment. This suggests that the blockage of CHK1 signaling enhances the efficacy of chemotherapic agents [119]. NVP-AUY922 also reduces the CHK1 levels to the sites of DNA damage in a dose-dependent manner, suppressing the CHK1-mediated G2 arrest in the presence of IR-induced DSBs [136].

#### 4.2.3. DNA-PK

The catalytic subunit of DNA-PK is a client of Hsp90 in the epithelioid cervix carcinoma cell line (HeLa), but not in normal the embryonic kidney human cell line (HEK293). Moreover, the cytosolic but not the nuclear DNA-PK levels are reduced in HeLa cells after treatment with the Hsp90 inhibitor radicicol [135]. The IR-induced activation of DNA-PK involves the interaction with ErbB1, which mediates DNA-PK transport into the nucleus [155]. Of note, human epithelial cells derived from pancreas carcinoma (MiaPaCa) exposed to 17-DMAG show the reduction of (i) the ErbB1 activity; (ii) the IR-induced interaction between ErbB1 and DNA-PK; and (iii) the IR-induced activation of DNA-PK, compromising the DSBs repair [60].

Hsp90α is phosphorylated extensively by DNA-PK at Thr5 and Thr7 in the nucleus of cells engaged to apoptosis. Phosphorylation is part of a signaling pathway activated by the TRAIL-induced apoptosis, by the Fas ligand, and by the apoptosis-inducer staurosporine [156,157]. An epigenetic landmark of early apoptosis is H2AX phosphorylation at the nuclear periphery of cells committed to apoptosis. This γ-H2AX nuclear staining is termed “the apoptotic γ-H2AX ring” that, although morphological distinct from the DDR, is characterized by the presence of several phosphorylated DDR proteins, such as ATM, NBN, CHK2, and DNA-PK [158,159]. Notably, the Thr5 and Thr7 phosphorylated form of Hsp90α also localizes into the apoptotic ring, where it acts as a modulator of the apoptotic process, enables the stabilization and activation of DNA-PK, and allows the execution of nuclear apoptosis [99,100]. Remarkably, Hsp90 inhibitors such as geldanamycin and 17-AAG enhance TRAIL-induced DNA-PK and H2AX activation [100,160].

Although both the DDR and the apoptotic ring are mediated by DNA-PK, the apoptosis-induced phosphorylation of Hsp90α at Thr5 and Thr7 residues differs from that induced from DDR. Indeed, the levels of the apoptosis-induced Hsp90α phosphorylation are markedly greater than those generated by DNA damage inducers [121]. Moreover, the apoptosis-induced Hsp90α phosphorylated form is distributed broadly and rapidly into the apoptotic ring, contrary to the much weaker, focally distributed, and delayed phosphorylation of Hsp90α in γ-H2AX foci formed during the DDR [99]. Finally, the apoptotic ring, unlike DDR foci, is defective for DNA repair because it lacks many DDR effector proteins, such as MDC1 and 53BP1. This is due to the fact that the caspase-mediated cleavage of MDC1, which normally binds to γ-H2AX and initiates the DDR, dissociates the FHA and BRCT domains of MDC1 and inactivates DNA repair in the apoptotic ring [158,161].

#### 4.2.4. The FA Pathway

The nuclear Fanconi Anemia (FA) “core complex” is involved in the FA pathway, also termed FA/BRCA pathway. This complex is composed of eight proteins including FANCA, FANCB, FANCC, FANCE, FANCF, FANCG, FANCL, and FANCM. FANCD2 and its paralogue/ binding-partner FANCI are activated by the ATR-mediated phosphorylation and by the FA core complex-dependent mono-ubiquitination [123]. Thus, the active forms of FANCD2 and FANCI localize to the chromatin at sites of DNA damage, where they interact with DNA repair proteins including BRCA1 and BRCA2. Indeed, the core complex and FANCD2 contribute in the maintenance of genome stability through the coordination of multiple DNA repair mechanisms, including HR, NHEJ, and translesion synthesis (TLS) [162,163].

Hsp90 regulates the FA pathway. Cell treatment with 17-AAG induces the rapid proteasomal degradation and cytoplasmic retention of FANCA, strongly suggesting that inhibition of Hsp90 induces nuclear depletion of the FA core complex impairing the activation of the FA pathway. Moreover, 17-AAG inhibits the DNA damage-induced activation of FANCD2, enhancing DNA cross-linker-induced cytotoxicity and chromosome aberrations [164,165].

#### 4.2.5. Histones

Changes in chromatin adjacent to the DNA break sites are instrumental to the DDR because they influence chromatin compaction and the binding of repair and signaling proteins to the DNA lesion [166]. Hsp90 interacts with high affinity with all histones; in particular, Hsp90 binding to the *C*-terminal tail of histone Hl influences chromatin remodeling, which represents a fundamental event in the DSB repair pathways [167]. Indeed, Hsp90 interferes with the regulatory Ser-Pro-Lys-Lys motifs of histone H1 impairing phosphorylation and acetylation during stress or steroid action(s) [168,169,170,171].

Of note, histone H1 has the unique feature to increase the Hsp90-associated Mg^2+^-dependent kinase activity 6- to 7-fold, which is not shared by other histones despite their similar high affinity for Hsp90. Furthermore, histone H1 has been reported to possess an ATP-binding activity regulating the ATP-turnover of Hsp90 [122].

#### 4.2.6. The MRE11/RAD50/NBN Complex

The treatment of MiaPaCa cells with 17-DMAG abrogates the IR-induced activation of the G2- and S-phase checkpoints, as well as DSB repair [54,60]. These effects seem to be the result of the disruption of the NBN/ATM axis, which in turn leads to a diminished activation of ATM after IR. Although both the levels of phosphorylated ATM and the number of phospho-ATM-containing nuclear foci were reduced in MiaPaCa cells after IR-induced damage, no interaction between ATM and Hsp90 has been detected. Although 17-DMAG neither induces degradation of any of the MRE11/RAD50/NBN components nor disrupts the MRE11/RAD50/NBN complex, nuclear Hsp90 binds the MRE11/RAD50/NBN complex. However, the exposure of MiaPaca cells to 17-DMAG compromises the ability of the MRE11/RAD50/NBN components to form nuclear foci and diminishes the interaction between NBN and ATM [60].

### 4.3. Other DNA Damage Response Clients

#### 4.3.1. MSH2

The DNA mismatch repair (MMR) pathway maintains genomic integrity by correcting DNA replication errors. MSH2 is a crucial protein involved in the highly conserved MMR mechanism, playing a pivotal role in the recognition of DNA mismatches and in the recruitment of other DNA repair proteins to the mismatched sites [172]. Under oxidative stress, both the p38 MAPK and c-Jun N-terminal kinase (JNK) pathways mediate the ectopic expression of MSH2 in renal carcinoma cell [173,174].

Anti-folates cause DSBs in human non-small-cell lung cancer (NSCLC) [175] and in human colon cancer cells [176]. The anti-folate pemetrexed possesses single-agent activity in front- and second-line treatments of NSCLC, especially adenocarcinoma. Pemetrexed causes a growth and survival response through the EGFR-mediated activation of the PI3K/AKT pathway. The combined treatment of NSCLC and lung adenocarcinoma cells with pemetrexed and with the 17-AAG Hsp90 inhibitor results in an enhanced pemetrexed-induced cytotoxic effect. In particular, the pemetrexed-induced down-regulation of the MKK3/6-p38 MAPK signal causes the reduction of MSH2 levels [174].

Tamoxifen, an estrogen receptor antagonist, has been used as therapy against breast cancer [177] and could promote cell death in NSCLC [178]. The combined treatment of tamoxifen with 17-AAG promotes the cytotoxic effect and growth inhibition of tamoxifen, significantly decreasing the expression of MSH2 in human lung carcinoma ‎cells treated with tamoxifen [179,180].

#### 4.3.2. PCNA and Polymerase η

TLS represents an essential mechanism for bypassing the block in the progress of replication forks. TLS is catalyzed by specialized polymerases, including the Y family polymerases. However, Y-polymerases display low stringency of the active site and a lack of proofreading, thus contributing to mutagenesis. Mammals have four members of Y-polymerases, *i.e.*, Pol η, Pol κ, Pol ι, and REV1 [181,182,183].

UV irradiation causes several types of DNA lesions, including cyclobutane pyrimidine dimers (CPDs) and (6–4) photoproducts ((6–4)PPs). Whereas (6–4)PPs are efficiently removed by the nucleotide excision repair pathway, CPDs frequently escape this mechanism. DNA polymerase η (Pol η) is a member of the mammalian Y family polymerases and performs error-free TLS across CPDs by incorporating correct bases on the opposite strand [184,185]. For this function, Pol η accumulates in nuclear foci at replication stalling sites via its interaction with monoubiquitinated PCNA [186,187]. The molecular chaperone Hsp90 promotes UV irradiation-induced nuclear focus formation of Pol η through control of its stability and binding to monoubiquitinated PCNA. Hsp90 facilitates the folding of Pol η into an active form in which PCNA- and ubiquitin-binding regions are functional. Furthermore, Hsp90 inhibition potentiates UV-induced cytotoxicity and mutagenesis in a Pol η-dependent manner, thus suggesting a role of Hsp90 as an essential regulator of Pol η-mediated TLS [188]. Of note, PCNA forms a complex with Hsp90 in HCT-116 (human colon cancer cell line), HT-29 (human colorectal adenocarcinoma), and in HNSCC-1483 (human head and neck cancer cell line), the treatment with geldanamycin resulting in the degradation of both PCNA and Hsp90 [189].

#### 4.3.3. XRCC1

Base modifications are perhaps the most common type of endogenous DNA damage, accounting for thousands of lesions per mammalian genome per day [190]. DNA alterations encompass alkylative and oxidative base products, abasic sites, strand breaks, and misincorporated nucleotides. The base excision repair (BER) pathway evolved to cope with the high level of spontaneous decay products that are formed in DNA, as well as those damages created upon reactions with natural endogenous chemicals, most notably reactive oxygen species. BER predominantly deals with non-bulky small nucleobase lesions, excising and replacing incorrect (*e.g.*, uracil) or damaged bases derived from deamination, alkylation or oxidation (*e.g.*, 3-methyladenine, 8-oxoG). The BER pathways can be divided in the following steps: (i) recognition and removal of an incorrect or damaged substrate base by a DNA glycosylase to create an abasic site intermediate; (ii) abasic site incision by an apurinic/apyrimidinic endonuclease or apurinic/apyrimidinic lyase; (iii) removal of the remaining sugar fragment by a lyase or phosphodiesterase; (iv) gap filling by a DNA polymerase; and (v) sealing of the nick by a DNA ligase [191]. In particular, upon damage, PARP1 activation triggers BER protein recruitment to facilitate short-patch or long-patch BER via DNA polymerase β (Polβ)-dependent or Polβ-independent mechanisms [192]. The heterodimer formed by XRCC1 and Polβ is necessary for the XRCC1-dependent recruitment of Polβ to the DNA damaged sites [193,194]. Overall, XRCC1 is an important scaffold protein that interacts with PARP1, DNA ligase III and Polβ to facilitate efficient repair of DNA single-strand breaks (SSBs) [195]. If SSBs are not properly repaired, they may be converted into DSBs during DNA replication, eventually leading to genetic instability and tumorigenesis [196]. Furthermore, down-regulation of XRCC1 expression in human breast cancer cell lines resulted in decreased SSB repair capacity and hypersensitivity to methyl methanesulfonate (MMS) [197]. PI3K/AKT pathway regulates the basal expression of XRCC1 in non-irradiated cells, and MKK1/2-ERK1/2 is essential for regulating induction of XRCC1 after radiation exposure [198].

Hsp90 protects the Polβ-unbound XRCC1 from degradation in a Polβ-binding-dependent manner [120]. By binding and stabilizing XRCC1, the phosphorylated form of Hsp90 promotes the formation of additional XRCC1 complexes. Indeed, in the absence of Hsp90 or following Hsp90 inhibition, free XRCC1 is removed by ubiquitin-mediated degradation, although also an undetermined ubiquitin-independent mechanism of XRCC1 degradation has been suggested [120].

Due to the varied and different DNA lesions induced by agents such as IR, H_2_O_2_ or cisplatin, the cellular response would likely require XRCC1-mediated repair that is both Polβ-dependent and/or Polβ-independent. Indeed, XRCC1 availability and involvement is crucial in BER sub-pathways but also in other repair processes such as NHEJ or nucleotide excision repair (NER) [199,200]. To date it has not been determined the mechanism through which cells regulate the Polβ/XRCC1 heterodimer nor the role for the Polβ-independent XRCC1. It has been suggested that the repair protein complex architecture for the regulation of such DNA repair processes, and in particular of the BER sub-pathway choice, depends upon the archetype BER complex (Polβ/XRCC1), the stabilized XRCC1/(phospho)-Hsp90 complex and XRCC1. Consistent with this model, complex formation (Polβ/XRCC1 *versus* XRCC1/Hsp90) varies in response to DNA damage or cell proliferation status. In particular, the XRCC1/Hsp90 or XRCC1/phospho-Hsp90 complexes might promote a role for XRCC1 in the DSB repair [199]. Therefore, the phospho-Hsp90- and proteasome-mediated regulation of Polβ and XRCC1 represent a mechanism of the DNA repair pathway choice that is likely to respond to cell type, cell cycle and exogenous stimuli, shedding light on Hsp90 as a novel functional component of BER that may facilitate BER sub-pathway choice in response to DNA damage and cellular proliferation [120].

Gefitinib is a selective epidermal growth factor receptor-tyrosine kinase inhibitor (EGFR-TKI) that blocks growth factor-mediated cell proliferation and ERK1/2 and AKT signaling activation. Furthermore, 17-AAG down-regulated the phospho-ERK1/2, phospho-AKT, and XRCC1 protein levels in gefitinib-exposed NSCLC cells. In addition, 17-AAG improved cell growth inhibition of gefitinib and potently reduced cell viability in both gefitinib-treated A549 and H1975 cell lines. These data provide a strong rationale for using an Hsp90 inhibitor in combination with EGFR-targeted therapies for the treatment of NSCLC [201].

## 5. Conclusions and Perspectives

Adaptation to stress is not only the driving force for species evolution but also a daily challenge that cells and organisms struggle against during their individual existence. An important mechanism for coping with stresses is the induction of molecular chaperones. These proteins, such as Hsp90, assist protein folding and protein complex assembly and act on misfolded proteins induced by stress conditions such as high levels of reactive oxygen species. Chaperone induction is a mode of adaption that does not involve genetic changes but rather reflects an evolved capacity to maintain proteostasis. The key role played by Hsp90 in the DNA damage sensing signaling, and repair, supported by the number of clients having a role in the DDR, leads to the hypothesis that genome instability arises when the demand for Hsp90 exceeds its functional capacity. Indeed, significant advances have been made recently in the elucidation of the molecular mechanisms of the DSBs damage response to IR, carcinogens, and environmental factors. The importance of a robust DNA-damage surveillance network is evidenced by the fact that defects in sensing, signaling and repair of DNA damage are linked to the development of inherited chromosome instability syndromes and cancer onset. The possibility to specifically interfere with the DSBs repair mechanisms for the development of novel therapeutics may have a significant clinical impact for the development of highly specific cancer therapies.

Cancer cells, which are presumed to be under higher stress conditions than normal cells, are characterized by increased Hsp90 chaperone expression. Furthermore, since Hsp90 and many co-chaperones undergo post-translational modifications upon cell stress, this may lead to biased assembly of Hsp90 complexes targeted to a subset of clients. The preferential chaperoning of some proteins over others could allow the Hsp90 machinery to restore homeostasis in a way tailored to a particular stress. However, a change in specificity implies that certain clients may no longer be chaperoned, thus causing genome instability.

Understanding the role of Hsp90 in genome stability has important implications for human disease. Although the susceptibility to disease may be genetically determined, a reasonable precept is that the actual transition to the disease state at the cellular level occurs when the assembly and plasticity of the encoded protein networks are compromised. Therefore, the primary function of the Hsp90 chaperone machinery may be to buffer against such transitions and, consequently, to disease. In light of these hypotheses, is reasonable to raise concerns about a therapeutic approach based solely on Hsp90 inhibition initially slowing tumor growth and ultimately favoring adaptation to chemotherapy. An alternative approach based on the simultaneous inhibition of Hsp90 and of proteasome-dependent protein degradation has been proposed. This may cause the complete loss-of-function and fast cell death, or simultaneously inhibiting Hsp90 and mitosis to prevent the emergence of chromosomally abnormal cell progeny. However, such drastic treatments are likely to increase unintended side effects on patients. The better understanding of Hsp90 client specificity and regulation under stress may enable more targeted approaches to prevent cancer cell proliferation as well as the potential for adaptive mutations.

## Abbreviations

17-AAG	17-(allylamino)-17-demethoxygeldanamycin
17-DMAG	17-dimethylaminoethylamino-17-demethoxygeldanamycin
BER	base excision repair
DDR	DNA damage response
DSB	DNA double-strand break
FA	Fanconi anemia
HR	homologous recombination repair
Hsp90	heat shock protein 90
IR	ionizing radiation
IRIF	IR-induced foci
MMR	DNA mismatch repair
NHEJ	non-homologous end-joining repair
NSCLC	non-small-cell lung cancer
Pol η	polymerase η
ssDNA	single-stranded regions of DNA
SSB	DNA single-strand break
TLS	translesion synthesis
