# Correction: Pennisi, R., et al. Hsp90: A New Player in DNA Repair? *Biomolecules* 2015, *5*, 2589–2618

**DOI:** 10.3390/biom6040040

**Published:** 2016-10-18

**Authors:** Rosa Pennisi, Paolo Ascenzi, Alessandra di Masi

**Affiliations:** 1 Department of Sciences, Roma Tre University, Viale Guglielmo Marconi 446, Roma I-00146, Italy; rosa.pennisi@uniroma3.it (R.P.); ascenzi@uniroma3.it (P.A.); 2 Istituto Nazionale di Biostrutture e Biosistemi, Viale Medaglie d’Oro 305, Roma I-00136, Italy

The authors of the published paper [1] wish to add the following two references on pages 2590 and 2597–2598 that were not originally cited in the text, consequently the paper has been updated:

11. Kaplan, K.B.; Li, R. A prescription for ‘stress’—The role of Hsp90 in genome stability and cellular adaptation. *Trends Cell Biol.* **2012**, *22*, 576–583.
105. Gullotta, F.; De Marinis, E.; Ascenzi, P.; di Masi, A. Targeting the DNA double strand breaks repair for cancer therapy. *Curr. Med. Chem.* **2010**, *17*, 2017–2048.

Furthermore, the following references were not correctly cited in the original paper, and have thus been updated:

122. Fang, Q.; Inanc, B.; Schamus, S.; Wang, X.H.; Wei, L.; Brown, A.R.; Svilar, D.; Sugrue, K.F.; Goellner, E.M.; Zeng, X.; et al. HSP90 regulates DNA repair via the interaction between XRCC1 and DNA polymerase beta. *Nat. Commun.* **2014**, *5*, doi:10.1038/ncomms6513.
198. Tung, C.L.; Jian, Y.J.; Syu, J.J.; Wang, T.J.; Chang, P.Y.; Chen, C.Y.; Jian, Y.T.; Lin, Y.W. Down-regulation of ERK1/2 and AKT-mediated *X*-ray repair cross-complement group 1 protein (XRCC1) expression by Hsp90 inhibition enhances the gefitinib-induced cytotoxicity in human lung cancer cells. *Exp. Cell Res.* **2015**, *334*, 126–135.

The authors apologize to the readers and authors of the cited papers for any inconvenience caused by these changes.
